# Distinct Roles of Molecular Chaperones HSP90α and HSP90β in the Biogenesis of KCNQ4 Channels

**DOI:** 10.1371/journal.pone.0057282

**Published:** 2013-02-19

**Authors:** Yanhong Gao, Sergey Yechikov, Ana E. Vazquez, Dongyang Chen, Liping Nie

**Affiliations:** Department of Otolaryngology, University of California Davis, Davis, California, United States of America; UMCG, The Netherlands

## Abstract

Loss-of-function mutations in the KCNQ4 channel cause DFNA2, a subtype of autosomal dominant non-syndromic deafness that is characterized by progressive sensorineural hearing loss. Previous studies have demonstrated that the majority of the pathogenic KCNQ4 mutations lead to trafficking deficiency and loss of KCNQ4 currents. Over the last two decades, various strategies have been developed to rescue trafficking deficiency of pathogenic mutants; the most exciting advances have been made by manipulating activities of molecular chaperones involved in the biogenesis and quality control of the target protein. However, such strategies have not been established for KCNQ4 mutants and little is known about the molecular chaperones governing the KCNQ4 biogenesis. To identify KCNQ4-associated molecular chaperones, a proteomic approach was used in this study. As a result, two major molecular chaperones, HSP70 and HSP90, were identified and then confirmed by reciprocal co-immunoprecipitation assays, suggesting that the HSP90 chaperone pathway might be involved in the KCNQ4 biogenesis. Manipulating chaperone expression further revealed that two different isoforms of HSP90, the inducible HSP90α and the constitutive HSP90β, had opposite effects on the cellular level of the KCNQ4 channel; that HSP40, HSP70, and HOP, three key components of the HSP90 chaperone pathway, were crucial in facilitating KCNQ4 biogenesis. In contrast, CHIP, a major E3 ubiquitin ligase, had an opposite effect. Collectively, our data suggest that HSP90α and HSP90β play key roles in controlling KCNQ4 homeostasis via the HSP40-HSP70-HOP-HSP90 chaperone pathway and the ubiquitin-proteasome pathway. Most importantly, we found that over-expression of HSP90β significantly improved cell surface expression of the trafficking-deficient, pathogenic KCNQ4 mutants L274H and W276S. KCNQ4 surface expression was restored by HSP90β in cells mimicking heterozygous conditions of the DFNA2 patients, even though it was not sufficient to rescue the function of KCNQ4 channels.

## Introduction

The voltage-gated potassium channel KCNQ4 plays a pivotal role in maintaining cochlear ion homeostasis and regulating hair cell membrane potential, both essential for normal auditory function [1]–[5]. Mutations in the KCNQ4 gene cause progressive sensorineural hearing loss in the DFNA2 patients [2], [3], [6]. To date, more than fifteen pathogenic KCNQ4 mutations have been identified and the majority of these mutations lead to decreased cell surface expression and loss of KCNQ4 currents [3], [7]–[21]. Despite the significance of the KCNQ4 biogenesis, little is known about the molecular mechanisms that control the process, which hinders the development of strategies to prevent and treat hearing loss of DFNA2 patients.

KCNQ4 channels contain six transmembrane domains, a pore-forming region, and two intracellular termini [22]. To become functionally active, KCNQ4 subunits must fold and assemble to tetrameric channels and translocate to specific locations on the plasma membrane [23]. In human cells, these processes are controlled by a sophisticated molecular chaperone network [24]. To identify molecular chaperones that are involved in KCNQ4 biogenesis, a proteomic analysis was first conducted in this study. As a result, two major molecular chaperones HSP70 and HSP90 were detected as KCNQ4-interacting proteins, suggesting that the biogenesis of the KCNQ4 channel may be controlled by the HSP90 chaperone pathway [24], [25]

Structural maturation of a HSP90 client requires the assistance of a specific set of chaperones and cochaperone. In most cases, HSP40, HSP70, and HOP are required in addition to HSP90 [24]–[27]. The requirement for a specific chaperone or cochaperone is client specific. For steroid hormone receptors, the most studied client of HSP90, cochaperone p23 is also required [25], [26]. In a current model, a newly synthesized polypeptide first interacts with HSP40 and HSP70 to form the initial receptor-chaperone complex; transfer of a receptor protein from HSP70 to HSP90 is facilitated by the cochaperone HOP, which is able to simultaneously bind HSP70 and HSP90 through separate tetratricopeptide repeat (TPR) domains. Binding of P23 to the chaperone complex promotes receptor maturation and dissociation of the complex after ATP hydrolysis [25], [26].

HSP90 is not required for de novo folding of most proteins, but it is essential for the structural maturation of a subset of proteins with multiple domains [24], including protein kinases, steroid hormone receptors, transcriptional factors, telomerase reverse transcriptase, endothelial nitric oxide synthase (eNOS) etc. [26]. The role of HSP90 in the biogenesis of membrane proteins, such as the cystic fibrosis transmembrane conductance regulator (CFTR), the ClC-2 chloride channel [28], the voltage-gated potassium channel hERG, and the ATP-sensitive potassium channel (K_ATP_) is also established [28]–[31]. Most importantly, recent studies have demonstrated that manipulating HSP90 function can be used to treat human diseases caused by misfolding and trafficking deficiency of mutant proteins [32]–[36].

In this study, we first verified that the KCNQ4 channel is a client protein of the molecular chaperone HSP90. Then, we tested whether HSP40, HSP70 and HOP, three key components of the HSP90 chaperone pathway, are required for the KCNQ4 biogenesis. In addition, we explored the potential of HSP40, HSP70, and HSP90 in rescuing surface expression of two trafficking-deficient DFNA2 mutants, L274H and W276S. Finally, we examined the effects of these mutations on the conductance of the KCNQ4 channel in HEK293T cells in which KCNQ4 surface expression was restored.

## Materials and Methods

### Chemicals and reagents

All chemicals were from Sigma-Aldrich (St. Louis, MO, USA); media and reagents for cell culture were from Invitrogen (Grand Island, NY, USA), unless otherwise indicated.

### Expression constructs

KCNQ4 (NM_004700) was cloned in pCMV6-XL5 vector and then tagged with a Myc or a modified HA epitope in the first extracellular loop of the KCNQ4 channel as described previously [15], [20]. These tagged KCNQ4 channels (referred as Myc-KCNQ4 or HA-KCNQ4) exhibited normal channel properties [15], [20]. Constructs of mutant KCNQ4 channels were generated from the tagged WT constructs using QuikChange Lighting Site-Directed Mutagenesis Kit (Stratagene, Santa Clara, CA, USA) and verified by DNA sequencing. For electrophysiological recordings, the wild type (WT) and mutant KCNQ4 channels were subcloned into pIRES2-DsRed2 vector. In addition, molecular chaperones HSC70 (heat-shock 70kD cognate protein, BC016179), HSP70 (BC063507), DJA1 (BC008182), and DJA2 (BC015809) were cloned in pCMV-SPORT6, while HSP90α (NM_005348) and HSP90β (NM_007355) were cloned in pCMV-script and pCMV6-XL5, respectively. Cochaperone HOP (heat-shock protein organizing protein, NM_006819) was cloned in pCMV6-XL5 and CHIP (C-terminal of HSP70-interacting protein, NM_005861.2) in pCMV6-AC.

### Antibodies

Primary antibodies used in this study were anti-HA (MMS-101P, Covance, Emeryville, CA, USA), anti-Myc (11667149001, Roche, Mannheim, Germany), anti-GAPDH (AM4300, Ambion, Austin, TX, USA), anti-HOP (H00010963-M01, Abonva, Taipei, Taiwan), anti-CHIP (#2080, Cell Signaling, Beverly, MA, USA), anti-HSP90α (ADI-SPS-771-F, Enzo Life Sciences, Plymouth Meeting, PA, USA), and those from Santa Cruz Biotechnology, Inc. (Santa Cruz, CA, USA), including anti-DjA1 (sc-59554), anti-DjA2 (sc-107520), anti-HSC70 (sc-1059), anti- HSP70 (sc-1060), and anti-HSP90 β (sc-1057). All of the horseradish peroxidase (HRP)-conjugated secondary antibodies were from Jackson ImmunoResearch Laboratories, Inc. (West Grove, PA, USA), including anti-goat (705-035-003), anti-mouse (715-035-151), and goat anti rabbit-HRP (711-035-003).

### siRNA

At least three different siRNA duplexes were used for each target RNA to ensure the knockdown effects were genuine. Shown here are the data from one representative duplex due to page limits. All experiments were done in parallel with non-specific controls. The following are sequences for all siRNAs used in this study - ***DJA1***: GUA CAU CAG CUC UCA GUA A, GAU CAG UCC UAA AGA UAG A, and GAA GCC AAU AUC UAC UCU U; ***DJA2***: GUA UCG UAA UCC CUU UGA A, CGU GAA GCC UAU AAU GAU A, and CCA UUG GCC UGA AGC UUA U; ***HSC70***: GAU CGA UUC UCU CUA UGA A, CCU UCG AGA UGC CAA ACU A, and CUG GAC AAG UGU AAU GAA A; ***HSP70***: GCA UCC CCA AGG UGC AGA A, CCU GUG UUU GCA AUG UUG A, CUG UGU UUG CAA UGU UGA A, and GUU GGU UAC UUC AAA GUA A; ***HSP90α***: CCC AGU UGA UGU CAU UGA UCA UCA A, CCC GUG AGA UGU UGC AAC AAA GCA A, and CCG AUU GGU GAC AUC UCC AUG CUG U; ***HSP90β***: CCC AGU UGA UGU CAU UGA UCA UCA A, CCC GUG AGA UGU UGC AAC AAA GCA A, and CCG AUU GGU GAC AUC UCC AUG CUG U; ***HOP***: GGG AGC UGA UAG AGC AGC UAC GAA A, AGG AAC CCG AAA GAU GCC AAA UUA U, and CAG AGA AUA AGA AGC AGG CAC UGA A; ***CHIP***: GGC AAU CGU CUG UUC GUG GGC CGA A, GGC AGU CUG UGA AGG CGC ACU UCU U, and CCA GCG CUC UUC GAA UCG CGA AGA A; ***Non-specific control***: CGA ACU CAC UGG UCU GAC Ctt and CGU GAU UGC GAG ACU CUG Att.

### Cell culture and transfection

HEK293T cells (Sigma-Aldrich) were used for all experiments. These cells were maintained according to the manufacturers’ instruction. All transfection were carried out using Lipofectamine 2000 as described by the manufacturer (Invitrogen). Following transfection, the cells transfected with plasmid DNA were incubated at 37 °C for 24 hrs, while those transfected with siRNA duplexes were cultured at 37°C for 48 hrs.

### Co-immunoprecipitation

The transfected cells were lysed in NP40 Lysis Buffer supplemented with protease inhibitor cocktail (P8340, Sigma-Aldrich), on ice for 30 min. Cell lysates were then cleared by centrifugation at 14,000 rpm for 10 mins at 4°C and incubated with primary antibodies as indicated at 4°C for 16 hrs. The protein complexes were isolated and purified using Dynabeads Protein G (100-04D, Invitrogen) following the manufacturer’s protocol and analyzed by Western blot.

### Isolation of surface KCNQ4 proteins

24 hrs post transfection, cells were washed twice with PBS *in situ* in 6-well culture plates and treated with mouse monoclonal anti-HA or anti-Myc antibodies (1:500 dilution) for 30 mins to label KCNQ4 channels on cell surface. Following three washes with phosphate buffered saline (PBS), the cells were lysed with NP40 lysis buffer supplemented with protease inhibitor cocktail (P8340, Sigma-Aldrich), on ice for 30 mins. The cell lysate was transferred to fresh tubes and centrifuged at 14,000 rpm for 10 mins at 4°C. Surface KCNQ4 proteins were captured using Dynabeads Protein G as instructed by the manufacturer. Proteins eluted from Dynabeads were analyzed by Western blot (see below).

### Western blot

Proteins were separated on Criterion^TM^ TGX Precast Gels (Bio-Rad Life Science, Hercules, CA, USA) and transferred to a nitrocellulose membrane (Bio-Rad Life Science). The membrane was probed with appropriate primary antibodies followed by incubation with HRP-conjugated secondary antibodies and visualized by SuperSignal West Pico Chemiluminescent Substrate (34077, Fisher Scientific). Chemiluminescent signals were collected by ChemiDoc XRP Imaging System and analyzed by Quantity One software (Bio-Rad Life Science). Each band was quantified as the total pixel value after subtraction of the background and normalized to the loading control protein GAPDH.

### Mass spectrometry analysis

Affinity purified proteins were trypsinized and concentrated. LC-MS/MS analysis was performed using a LTQ linear ion trap mass spectrometer (Thermo Scientific) in conjunction with a Paradigm MG4 HPLC (Michrom Bio Resources) at the Proteomics Center of University of California, Davis (http://proteomics.ucdavis.edu/). For peptide identification, the Uniprot reference human proteome database (March, 2012) was used. Scaffold (Proteome Software) was used to validate MS/MS based peptide and protein identifications. Peptide and protein identifications were only accepted if they could be established at greater than 95% and 95% probability respectively [37], and contained at least two identified peptides matched per protein entry.

### Whole-cell voltage clamp

HEK293T cells were trypsinized 24 hrs after transfection, seeded onto poly-L-lysine coated glass coverslips, and maintained under normal growth condition for about 4 hrs. Before recording, cells were extensively washed with external solution (10 mM NaCl, 4.5 mM KCl, 2 mM CaCl2, 1 mM MgCl2, 10 mM HEPES, pH 7.4, and osmolarity of 303 mmol/kg). Only healthy looking attached cells expressing DsRed fluorescent marker were used for recordings. Glass electrodes with resistance ranging from 1.5 to 3.0 MΩ and filled with internal solution (2.5 mM Na2ATP, 135 mM KCl, 3.5 mM MgCl2, 5 mM EGTA, 2.41 mM CaCl2, 5 mM HEPES pH7.2, and osmolarity of 300 mmol/kg) were used. Data acquisition was performed using a HEKA EPC-10 amplifier and HEKA PatchMaster software (HEKA, Bellmore NY) with high band Bessel filter set to 10 kHz and low band filter set to 0.2 kHz. The protocol consisted of a 1 sec -80 mV interval, then depolarization from -80 to +50 mV in 10 mV steps for 1.5 sec, then -50 mV for 1 sec followed by 1sec at -80 mV, with 5 sec in between testing sequences. Representative whole-cell current traces were plotted using Origin v8.6 software (OriginLab Corp, Northampton, MA).Whole-cell current densities (pA/pF) were calculated as the maximal current (pA) divided by the cell capacitance, C-slow (pF). Each day at least 5 cells expressing WT KCNQ4 were recorded as internal control group. All recordings were performed at room temperature.

### Statistics

Biochemical results were presented as mean ± SD of at least three independent experiments; while electrophysiological data were presented as mean ± SEM of whole-cell current densities from 5-30 cells. The measurements were statistically analyzed using two-tailed unpaired Student’s t test (MS Excel 2010). Significance was reported as *p ≤ 0.05 and **p ≤0.01.

## Results and Discussion

### Identification of Molecular Chaperones Associated with KCNQ4 Channels

HA-KCNQ4 was expressed in HEK293T cells. Proteins associated with KCNQ4 subunits were affinity purified and identified by LC-MS/MS. The experiment was conducted in triplicate and in parallel with three negative control experiments in which HEK293T cell were transfected by vector DNAs. HSP70 was identified in all three replicates, while HSP90 was detected in one of three individual experiments. Specifically, a total 31 unique spectrums were detected for HSP70, representing 48% sequence coverage. On the other hand, only two unique spectrums were identified for HSP90, corresponding to 5% of the amino acid sequence common to both the inducible HSP90α and the constitutive HSP90β. Since interactions between molecular chaperones and their clients are highly dynamic [26], [38], we considered both HSP70 and HSP90 potential KCNQ4 interacting proteins. In addition, we previously identified HSP40 as a KCNQ4 interacting protein in a yeast-two-hybrid screen. These data suggested that the KCNQ4 biogenesis might be controlled by the HSP40-HSP70-HSP90 chaperone pathway.

### Roles of HSP90α and HSP90β in KCNQ4 Biogenesis

**Figure 1:**
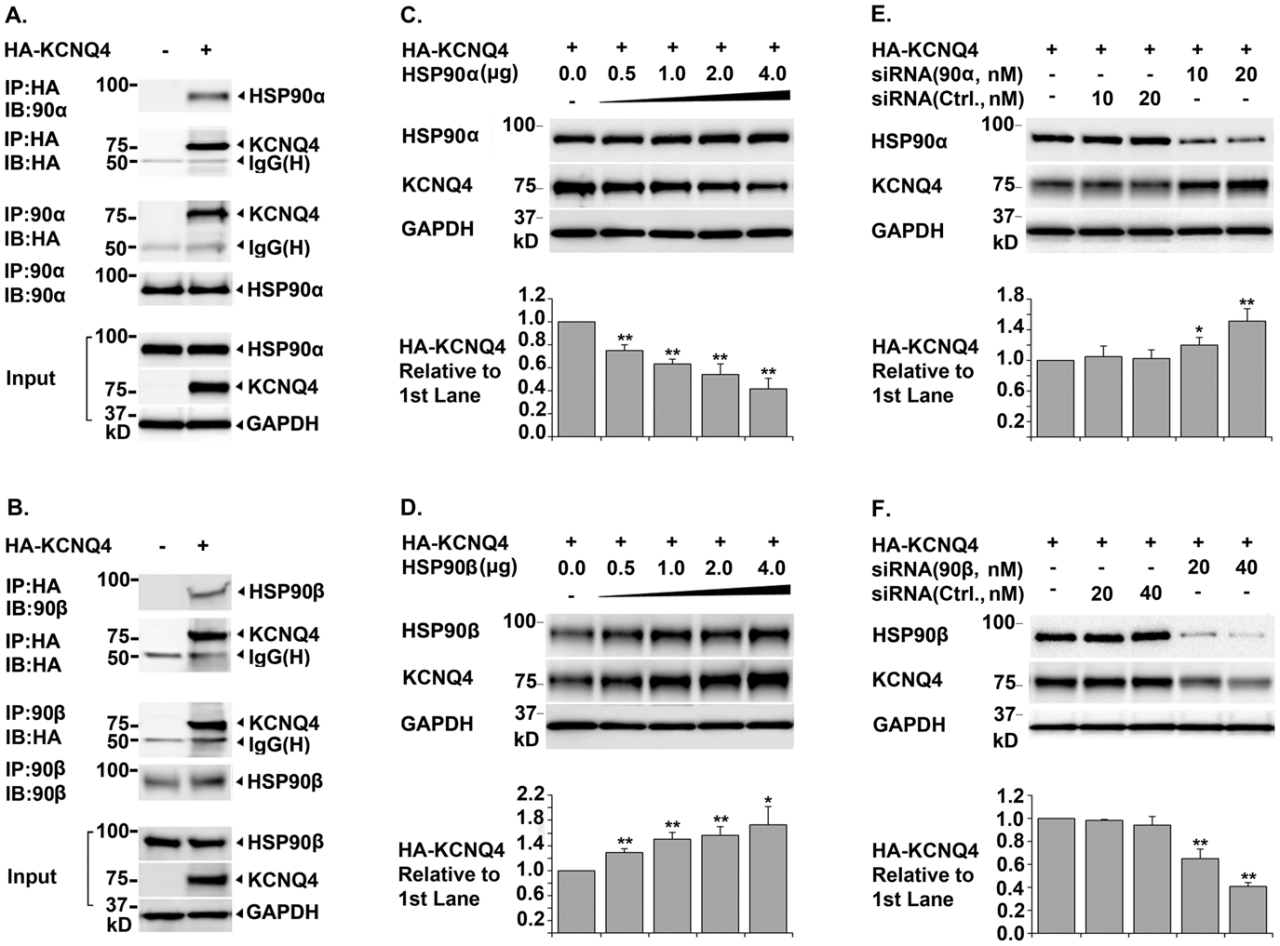
Opposite Roles of Molecular Chaperones HSP90α and HSP90β in KCNQ4 Biogenesis. *A. & B. Co-immunoprecipitation of KCNQ4 channels with the molecular chaperones HSP90 & HSP90β* HEK293T cells were transfected with 0.4 µg of pCMV6-XL5-HA-KCNQ4 (+) or pCMV6-XL5 alone (-) and harvested 24 hrs post transfection. Immunoprecipitation (IP) was carried out respectively using a mouse monoclonal antibody against the HA tag in the first extracellular loop in KCNQ4 channels or an antibody against HSP90α (or HSP90β). Precipitated proteins were then analyzed by Western blot (IB) using the indicated antibodies; cell lysates were tested in parallel as input controls. Both HSP90α and HSP90β were co-immunoprecipitation with the KCNQ4 channels and vice versa, indicating that these two chaperones are associated with KCNQ4 channels. *C. & D. Effects of over-expression of HSP90α or HSP90β on total KCNQ4 level* HEK293T cells were transfected with 0.4 µg of pCMV6-XL5-HA-KCNQ4 (+) and various amount of HSP90α or HSP90β cDNA as indicated. The cells were then lysed in NP40 lysis buffer 24 hrs post transfection and the total amount of KCNQ4 proteins in the cell lysates were assessed by Western blot. Over-expression of HSP90β resulted in a significant increase, while over-expression of HSP90α led to a marked decrease in total KCNQ4 level. *E. & F. Effects of siRNA knockdown of HSP90α or HSP90β on total KCNQ4 level* HEK293T cells were transfected with 0.4 µg of pCMV6-XL5-HA-KCNQ4 cDNA (+) and various amount of siRNA specifically targeting HSP90α or HSP90β as indicated. Non-specific siRNA (Ctrl.) were used in parallel as negative controls. Total KCNQ4 proteins were assessed by Western blot 48 hrs post transfection. Specific knockdown of HSP90β led to a dramatic decrease, whereas knockdown of HSP90α resulted in a marked increase in total KCNQ4 level. Each data point in the bar graphs represents the mean ±SD of three experiments (* p≤0.05, ** p≤0.01).

To determine whether HSP90 is involved in KCNQ4 biogenesis, we first conducted reciprocal co-immunoprecipitation assays in transfected HEK293T cells to confirm the interaction between KCNQ4 and two HSP90 isoforms. Western blot showed that both HSP90α and HSP90β co-precipitated with KCNQ4 proteins; conversely, KCNQ4 proteins were detected in HSP90α and HSP90β immunoprecipitates (Figure 1, A. and B.), which confirmed that HSP90α and HSP90β physically interact with KCNQ4 proteins. Next, we examined the effects of HSP90α or HSP90β knockdown on the total amount of the KCNQ4 channel (**total KCNQ4 level**), using siRNAs specifically targeting HSP90α or HSP90β. Surprisingly, we found that knockdown of two different HSP90 isoforms had opposite effects on total KCNQ4 level. Specifically, knockdown of HSP90β led to a dramatic decrease; while knockdown of HSP90α resulted in a marked increase in total KCNQ4 level (Figure 1, E. and F.). Consistent with these results, over-expression of HSP90β increased; whereas up-regulation of HSP90α expression decreased total KCNQ4 level (Figure 1, C. and D.).

HSP90α and HSP90β are closely related isoforms with approximately 85% identity at the amino acid level. The functions of the two isoforms are highly overlapped but not completely redundant [26], [39]. Isoform specific functions were reported previously. For example, extracellular HSP90α, but not HSP90β, is involved in cancer cell invasion, angiogenesis, and metastasis [40]–[43]. Similarly, HSP90α rather than HSP90β is required for the maturation and trafficking of the voltage-gated potassium channel hERG [30]. Moreover, opposite effects of HSP90α and HSP90β on the activities of endothelial nitric oxide synthase have been reported in a recent study [44]. The two isoforms of HSP90 differentially regulate phosphorylation of eNOS and therefore have different effects the conformation and activity of the enzyme [44]. Consequently, over-expression of HSP90α resulted in a significant increase in NO_2_/NO_3_ levels, whereas over-expression of HSP90β led to reduced NO_2_/NO_3_ production [44]. These findings together improved our understanding of isoform differences of HSP90.

### Requirements of HSP40, HSP70, and HOP in KCNQ4 Biogenesis

**Figure 2:**
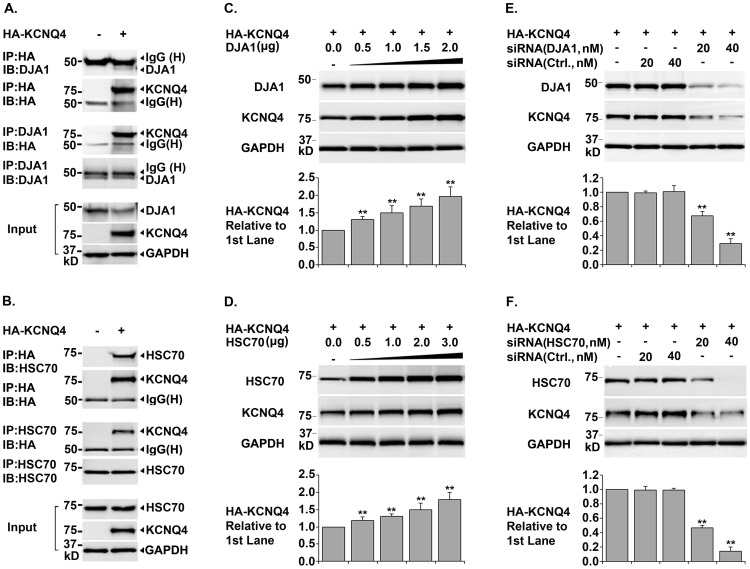
Roles of HSC70 and Cofactor DjA1 in KCNQ4 Biogenesis. *A. & B. Co-immunoprecipitation of KCNQ4 channels with HSP70 or DJA1* HEK293T cells were transfected with 0.4 µg of pCMV6-XL5-HA-KCNQ4 (+) or pCMV6-XL5 (-) and cultured for 24 hrs. Immunoprecipitation (IP) was carried out respectively using a mouse monoclonal antibody against the HA tag in KCNQ4 channels or an antibody against HSC70 (or DJA1). Precipitated proteins were detected by Western blot (IB) using the indicated antibodies. Cell lysates were also tested for HA-KCNQ4, HSC70, and GAPDH as input controls. Both HSP70 and DJA1 were precipitated with KCNQ4 channels, and vice versa. *C. & D. Effects of over-expression of HSC70 or DjA1 on total KCNQ4 level* HEK293T cells were transfected with 0.4 µg of pCMV6-XL5-HA-KCNQ4 (+) and various amount of each chaperone cDNA as indicated. The transfected cells were cultured for 24 hrs and KCNQ4 proteins in the cell lysates were assessed by Western blot. Over-expression of either HSC70 or DJA1 induced a dramatic increase in total KCNQ4 level. *E. & F. Effects of siRNA knockdown of HSC70 or DjA1 on total KCNQ4 level* HEK293T cells were transfected with 0.4 µg of pCMV6-XL5-HA-KCNQ4 (+) and various amount of siRNA specifically targeting HSC70 or DjA1 as indicated. Non-specific siRNA (Ctrl.) were transfected in parallel as negative controls. 48 hrs post transfection, the cell lysates were collected and analyzed for total KCNQ4 proteins. Knockdown of HSC70 or DJA1 resulted in a significant decrease in total KCNQ4 level. Each data point in the bar graphs represents the mean ±SD of three experiments (* p≤0.05, ** p≤0.01).

**Figure 3:**
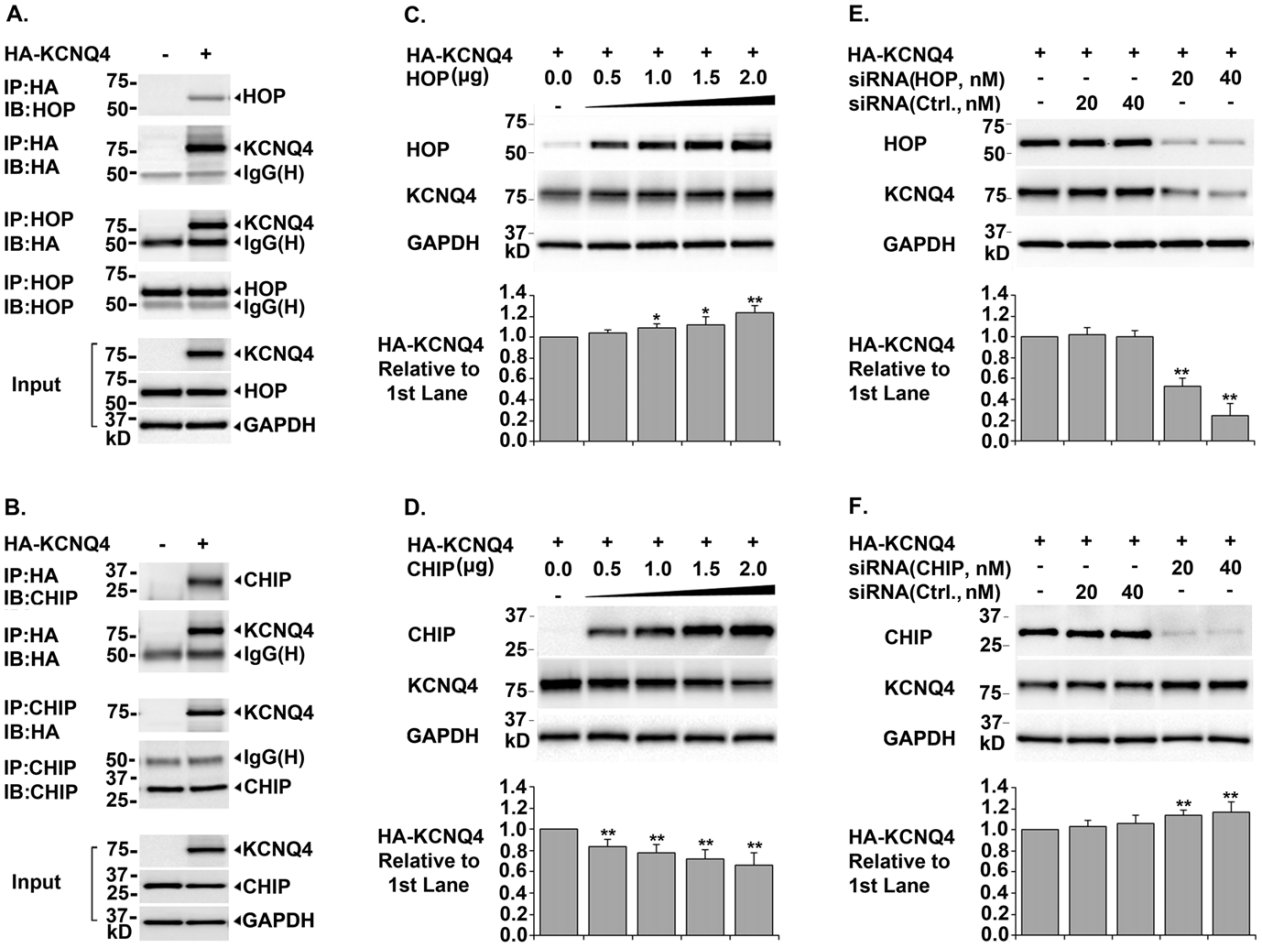
Roles of Cochaperones HOP and CHIP in KCNQ4 Biosynthesis. *A. & B. Co-immunoprecipitation of KCNQ4 channels with cochaperones HOP and CHIP* HEK293T cells were transfected with 0.4 µg of pCMV6-XL5-HA-KCNQ4 (+) or pCMV6-XL5 (-) and cultured for 24 hrs. Immunoprecipitation (IP) was done using antibodies either against the HA tag in KCNQ4 channels or a cochaperone (HOP or CHIP). The precipitate was analyzed by Western blot (IB) using the indicated antibodies. Cell lysates were tested in parallel for HA-KCNQ4, cochaperones, and GAPDH as input controls. Association of HOP and CHIP with KCNQ4 channels were confirmed by co-precipitation of these proteins. *C. & D. Effects of over-expression of HOP or CHIP on total KCNQ4 level* HEK293T cells were transfected with 0.4 ug of pCMV6-XL5-HA-KCNQ4 (+) and different amount of cochaperone cDNA as indicated. 24 hrs post transfection, the cells were lysed in NP40 lysis buffer and the cell lysates were tested for total KCNQ4 proteins by Western blot. Over-expression of cochaperone HOP led to a significant increase, whereas over-expression of CHIP resulted in a marked decrease in total KCNQ4 level. *E. & F. Effects of siRNA knockdown of HOP or CHIP on total KCNQ4 level* HEK293T cells were transfected with 0.4 µg of pCMV6-XL5-HA-KCNQ4 (+) and various amount of siRNA as indicated. The transfected cells were cultured for 48 hrs and the cell lysates were tested for KCNQ4 proteins. Knockdown of cochaperone HOP led to a marked decrease, while knockdown of CHIP resulted in a significant increase in total KCNQ4 level. Each data point in the bar graphs represents the mean ±SD of three experiments (* p≤0.05, ** p≤0.01).

Three lines of experiments were conducted to test whether these key components of the HSP90 chaperone pathway were required for KCNQ4 biogenesis. Both the constitutive and inducible isoforms of HSP40 and HSP70 were tested. Co-immunoprecipitation data showed that DjA1 and HSC70, the constitutive isoforms of HSP40 and HSP70, were physically associated with KCNQ4 subunits (Figures 2, A. and B.). Over-expression of DjA1 or HSC70 considerably increased total KCNQ4 level; while siRNA knockdown of these chaperones significantly reduced total KCNQ4 level (Figure 2, C. D. E. and F.). Similarly, manipulating expression of DjA2 and HSP70, the inducible isoforms of HSP40 and HSP70, had the same effects on total KCNQ4 level (data not shown), indicating that HSC70 and HSP70 are functionally interchangeable in promoting KCNQ4 biogenesis; so are the cofactors DjA1 and DjA2. Using similar experimental strategies, we also confirmed that the cochaperone HOP is essential for KCNQ4 biogenesis (Figure 3, A. C. and E.). Collectively, these data demonstrated that HSP40, HSP70, and HOP are required for the KCNQ4 biosynthesis.

### CHIP is involved in Degradation of KCNQ4 Channels

HSP90 plays a well-established role in assisting proteins to achieve and maintain their active conformations [24], [26], [39]. It has recently become clear that HSP90 also plays a key role in the triage of aberrant, misfolded, or damaged proteins for degradation via the ubiquitin-proteasome pathway [45], [46]. In addition, CHIP was found as a major E3 ubiquitin ligase for HSP90 client proteins [26], [45], [46]. Our results support the role of the cochaperone CHIP in promoting degradation of KCNQ4 channels (Figure 3, B. D. and F.). First, co-immunoprecipitation assays showed that CHIP is a KCNQ4-interacting protein. Secondly, CHIP over-expression caused a marked decrease in total KCNQ4 level. Lastly, siRNA knockdown of CHIP led to a considerably increased total KCNQ4 level.

### HSP90α and HSP90β - Key Players in Maintaining KCNQ4 Homeostasis

Mechanisms that control the balance between biosynthesis and degradation of HSP90 client proteins are not yet well-understood. However, advances were made in a recent study [47]. Kundrat and Regan demonstrated that HSP90 client proteins have much higher probability for folding than degradation under normal growth conditions; when a client protein cannot be folded properly due to HSP90 inhibition, a genetic mutation, or a cellular stressor, it will have a greater chance to interact with HSP70-CHIP complexes and therefore to be selected for ubiquitin-dependent degradation [45], [47]. Based on these findings and our data in this study, we propose the following hypothesis. Under normal growth condition, when the constitutive HSP90β is the major HSP90 isoform, the folding and maturation of KCNQ4 channels are mainly via the HSP40-HSP70-HOP-**HSP90β** pathway. However, when HSP90α expression is elevated due to induction or heterologous expression, HSP90α replaces HSP90β in the HSP90 chaperone pathway and the structural maturation of KCNQ4 channels would proceed through the HSP40-HSP70-HOP-**HSP90α** pathway, in which KCNQ4 channels is more likely to associate with HSP70-CHIP complexes and to be targeted for degradation via ubiquitination-proteasome pathway. Thus, the relative abundance of HSP90α and HSP90β is a key factor controlling KCNQ4 homeostasis.

### Effects of HSP40, HSP70, and HSP90β on KCNQ4 Surface Expression

**Figure 4:**
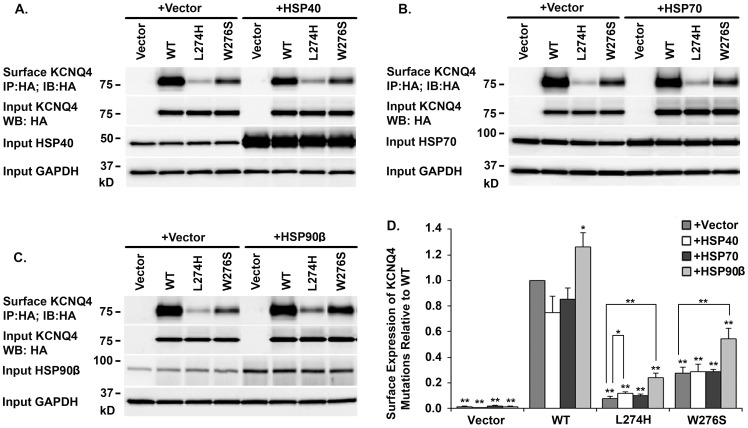
Effects of HSP40, HSP70, and HSP90β on KCNQ4 Surface Expression. *A. B. & C. Representative Western bolt* HEK293T cells were transfected with indicated combination of plasmid DNAs (0.4 µg of a KCNQ4 channel and 1 µg of a chaperone or a vector as indicated on the top of the blots). 24 hrs post transfection, KCNQ4 channels on cell surface were labeled with a mouse monoclonal antibody against the HA tag in KCNQ4 channels prior to cell lysis, then, isolated using Dynabeads Protein G (IP) and assessed by Western blot (IB). ***D. Summary data*** Each data point in the bar graphs represents the mean ±SD of three experiments (* p≤0.05, ** p≤0.01). Compared with HSP40 and HSP70, HSP90β showed the highest rescue efficiency of KCNQ4 mutants.

Three key components in the HSP90 chaperone pathway were co-expressed individually with the WT KCNQ4 or a mutant channel (L274H or W276S) in HEK293T cells. KCNQ4 channels on cell surface were affinity purified and analyzed by Western blot (Figure 4). Over-expression of HSP90β significantly increased surface expression of WT KCNQ4 channels (26 ± 10.93%), while over-expression of HSP40 and HSP70 had no effect (Figure 4). In addition, over-expression of HSP40 induced a mild increase in cell surface expression of the mutant L274H, but not W276S (Figure 4, A and D). Similarly, up-regulation of HSP70 expression had no significant effects on surface expression of either mutant (Figure 4 B and D). The highest rescue efficiencies were achieved by over-expression of HSP90β, which led to more than two fold increases in cell surface expression of both mutants (Figure 4 C and D).

### Rescue of KCNQ4 Mutants in Cells Mimicking Heterozygous Condition of DFNA2 Patients

**Figure 5:**
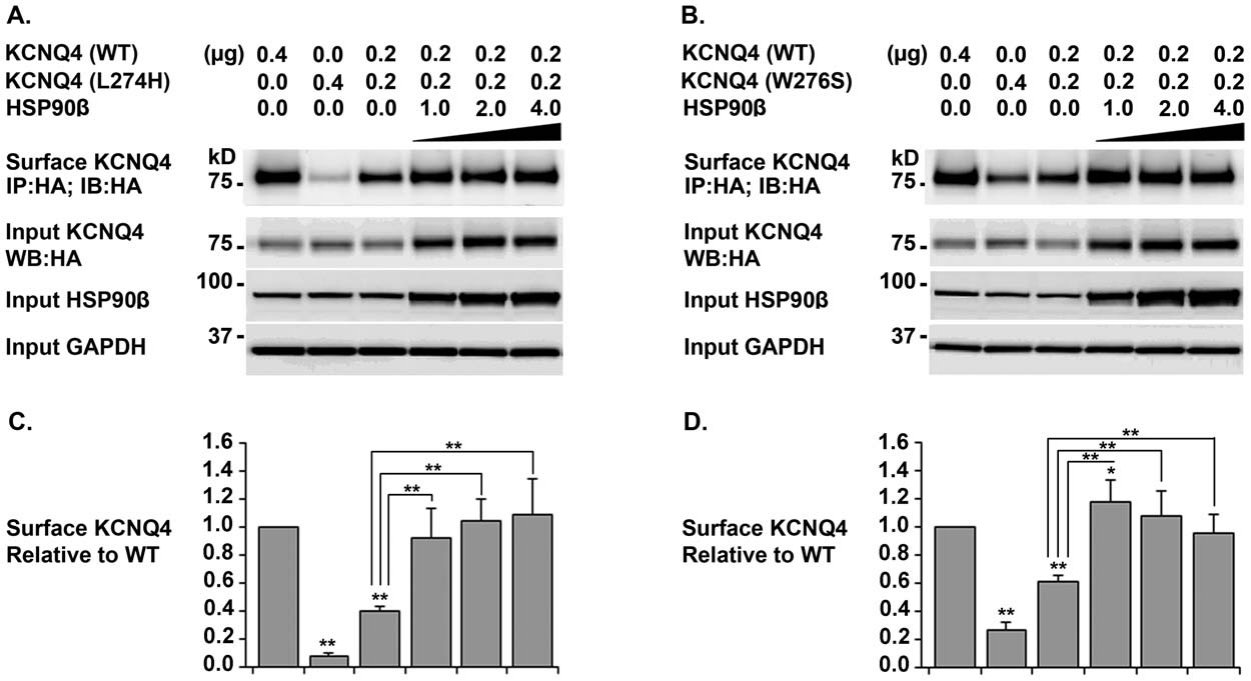
Rescue KCNQ4 Surface Expression in Cells Mimicking the Heterozygous Condition of DFNA2 Patients. *A. & B. Representative Western blot* HEK293T cells were transfected with WT and a mutant KCNQ4 channel at a ratio 1:1 plus various amount of HSP90β as indicated. 24 hrs post transfection, KCNQ4 channels on cell surface were labeled with a mouse monoclonal antibody against the HA tag in the first extracellular loop of KCNQ4 channels prior to cell lysis; then isolated using Dynabeads Protein G and analyzed by Western blot. *C. & D. Summary data* Each data point in the bar graphs represents the mean ±SD of three experiments (* p≤0.05, ** p≤0.01). Surface expression in cells co-expressing WT/L274H or WT/W276S could be restored to the level of WT KCNQ4 by over-expression of HSP90β.

**Figure 6:**
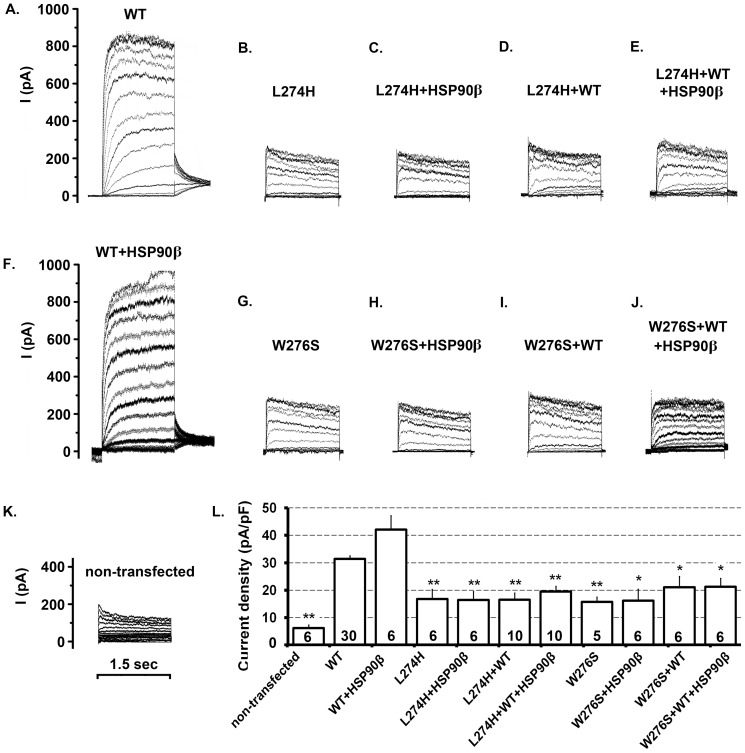
Functional Consequences of Restoration of KCNQ4 Surface Expression. *A-K. Representative raw traces* Whole-cell currents were recorded from transfected HEK293T cells. **A.** 0.5 µg WT KCNQ4 + 2.5 µg vector; **B.** 0.5 µg L274H + 2.5 µg vector; **C.** 0.5 µg L274H + 2.5 µg HSP90β; **D.** 0.25 µg L274H + 0.25 µg WT KCNQ4 + 2.5 µg vector; **E.** 0.25 µg L274H + 0.25 µg WT KCNQ4 + 2.5 µg HSP90β; **F.** 0.5 µg WT KCNQ4 + 2.5 µg HSP90β; **G.** 0.5 µg W276S + 2.5 µg vector; **H.** 0.5 µg W276S + 2.5 µg HSP90β; **I.** 0.25 µg W276S + 0.25 µg WT KCNQ4 + 2.5 µg vector; **J.** 0.25 µg W276S + 0.25 µg WT KCNQ4 + 2.5 µg HSP90β; **K.** non-transfected cells. ***L.*** ** ***Average whole-cell current densities*** **(pA/pF)** were calculated as the maximal current (pA) divided by the cell capacitance (pF). Each data point in the bar graph represents the average ± SEM of the indicated number of cells and was compared to the average current density of the WT channel (* p≤0.05, ** p≤0.01). The time scale on **K.** applies to all panels.

The WT KCNQ4 channel was co-expressed with a mutant channel at a ratio of 1 to 1 to mimic the heterozygous condition of the DFNA2 patients. Surface KCNQ4 channels were isolated by affinity purification from HEK293T cells expressing WT/L274H+HSP90β and those expressing WT/W276S+HSP90β. In both cases, Western blot showed that KCNQ4 surface expression could be restored to the WT level by HSP90β over-expression (Figure 5). In addition, to assess the functional effects of HSP90 over-expression, whole-cell currents were recorded under similar conditions. No significant improvement in average current density was observed in HEK293T cells in which KCNQ4 surface expression was restored (Figure 6). Specifically, the average current density was 31.37 ± 1.20 pA/pF (n = 30) for the WT alone and 42.07 pA/pF ±5.13 (n = 6) for WT/HSP90β, but 16.81 ± 3.45 pA/pF (n = 6) for L274H, 16.46 pA/pF ±3.06 (n = 6) for L274H/HSP90β, 16.56 ± 2.40 pA/pF (n = 10) for WT/L274H, and 19.46 ± 2.0 pA/pF (n =  10) for WT/L274H/HSP90β (Figure 6, A–F and L). Moreover, the average current density was 15.77 ± 1.71 pA/pF (n = 5) for W276S, 16.13 pA/pF ±4.20 (n = 6) for W276S/HSP90β, 21.04 ± 4.0 pA/pF (n = 6) for WT/W276S, and 21.21 ± 3.12 pA/pF (n =  6) for WT/W276S/HSP90β, while it was 6.31 ± 1. 01 pA/pF (n = 5) for non-transfected cells (Figure 6, G–L). Therefore, our data demonstrated that the restoration of cell surface expression do not lead to the functional rescue of these two KCNQ4 mutants, indicating that both L274H and W276S disrupt not only surface expression but also the conductance of the KCNQ4 channel.

In this study, we demonstrated that the biogenesis of the KCNQ4 channel is controlled by the HSP40-HSP70-HOP-HSP90 chaperone pathways. The cellular level of the KCNQ4 channel depends on the relative abundance of HSP90α and HSP90β, which have opposite effects on KCNQ4 biosynthesis. HSP90 has recently emerged as a promising drug target for the treatment of cancer and neurodegenerative diseases caused by protein misfolding [26], [42], [48]–[53]; comprehensive understanding of the isoform difference of HSP90 is fundamental for the rational design of specific and effective therapeutic interventions. We also showed that cell surface expression of the KCNQ4 channel can be restored to the normal level in the cells mimicking the heterozygous conditions of DFNA2 patients, even though the restoration of surface expression did not lead to functional rescue. L274H and W276S are located in the pore helix of the KCNQ4 channel; our data confirm that both L274 and W276 are critical in maintaining the integrity of the K+-permeable pore and KCNQ4 functions. The findings in this study therefore laid important groundwork for future research towards functional rescue of KCNQ4 mutants and hearing restoration of DFNA2 patients.
